# Zoonotic sporotrichosis in pediatric patients: analysis of 30 cases at a referral center in São Paulo, Brazil^؆^

**DOI:** 10.1016/j.abd.2026.501346

**Published:** 2026-04-23

**Authors:** Bruna Cavaleiro de Macedo Souza, Guilherme Camargo Julio Valinoto, John Verrinder Veasey

**Affiliations:** a Faculdade de Ciências Médicas, Santa Casa de Misericórdia de São Paulo, São Paulo, SP, Brazil; b Dermatology Clinic, Hospital da Santa Casa de Misericórdia de São Paulo, Santa Casa de Misericórdia de São Paulo, São Paulo, SP, Brazil

Dear Editor,

Sporotrichosis is a subcutaneous and/or systemic mycosis caused by fungi of the genus *Sporothrix*, whose epidemiology has undergone significant changes in recent decades in Latin America, due to the increase in cases of zoonotic transmission by infected cats, mainly the species *S. brasiliensis*.^1^, ^2^, ^3^, ^4^ In this new scenario, a change in the profile of the most affected hosts has been observed, with a higher incidence in individuals at the extremes of age, such as the elderly and children, who maintain frequent contact with sick animals.^4^, ^5^ Despite the increase in cases in the pediatric population, which has its own clinical-epidemiological characteristics, studies focused on this group are still scarce.

Given this gap, a retrospective study was conducted analyzing the medical records of pediatric patients diagnosed with sporotrichosis at a referral center in the city of São Paulo, Brazil, between 2012 and 2024. The study was approved by the institution's Research Ethics Committee (CAAE: 86929625.0.0000.5479).

The inclusion criterion was a confirmed diagnosis of sporotrichosis in patients under 18 years of age during the aforementioned period. A total of 30 patients were included, aged between one and 16 years (mean 8.7 years). Table 1 shows their clinical and epidemiological characteristics. The most prevalent clinical forms of sporotrichosis in this study are shown in Fig. 1. The diagnosis was clinical-epidemiological in 20 cases (66.7%) and clinical-laboratory in 10 (33.3%). Laboratory confirmation was performed by isolating the fungus in culture in 24 patients (80%) and/or by histopathological examination with Grocott/PAS staining in 19 patients (63%). Table 2 summarizes the treatment instituted and the clinical evolution of the cases.

**Table 1:** Clinical, epidemiological, and laboratory characteristics of pediatric patients with sporotrichosis.

Variable	Category	n (%)
Sex	Female	16 (53.3%)
	Male	14 (46.7%)
Location	Face	14 (46.6%)
	Upper limbs	11 (36.6%)
	Trunk and cervical region	3 (10%)
	Lower limbs	2 (6.6%)
Clinical form	Cutaneous-lymphatic	17 (56.7%)
	Localized cutaneous	4 (13.3%)
	Multiple inoculations	4 (13.3%)
	Ocular mucosa	4 (13.3%)
	Immunoreactive	1 (3.3%)
Contact with feline	Yes	26 (86.6%)
Total number of cases	‒	30 (100%)
	No	4 (13.3%)
Isolation of *Sporothrix spp*. in culture	Positive	18 (75%)
	Negative	6 (25%)
	Tests performed	24 (100%)
Evidence of yeast in Grocott-PAS	Positive	4 (21%)
	Negative	15 (79%)
	Tests performed	19 (100%)

**Fig. 1:**
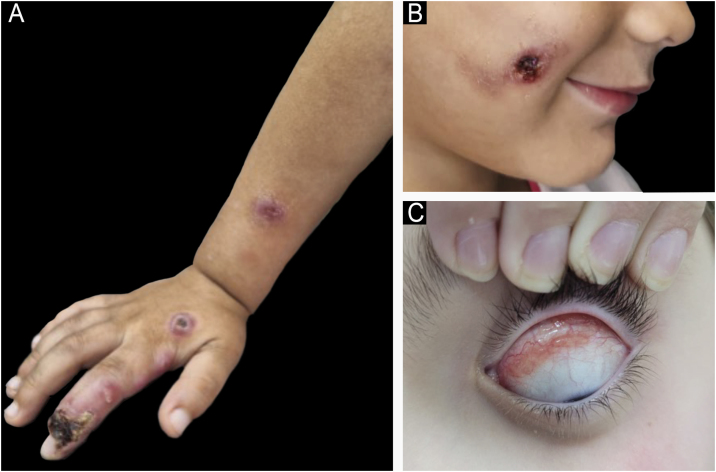
Clinical presentation of sporotrichosis in children. (A) Cutaneous-lymphatic form. (B) Localized cutaneous form. (C) Mucosal (ocular) form.

**Table 2:** Treatment profile and clinical outcomes.

Medications used in the treatment	30 patients (100%)	Dose	Average treatment time until cure (months)	Cure (cure rate)	Loss to follow-up (number of cases)
Itraconazole	19 (63%)	100 mg day	3	13 (68.4%)	6
Potassium iodide	11 (37%)	100 mg kg.day	5	9 (81.8%)	2

The findings can be analyzed from two perspectives: in comparison to data from the adult population affected by the same mycosis, and in relation to pediatric data already described in the literature, both for the *S. schenckii* complex and the zoonotic clade *S. brasiliensis*.

The anatomical distribution of the lesions showed a predominance on the face, followed by the upper limbs. This pattern may be related to the characteristic behavior of children of petting animals close to their faces.^3^, ^4^ While an analysis of the general population in São Paulo (including all age groups) showed a higher frequency of lesions in the upper limbs (78.8%),^3^ the importance of the face as an affected site in children during zoonotic outbreaks has already been reported, with reports of facial lesions in up to 50% of pediatric cases.^6^

The Lymphocutaneous (LC) form was the most frequent clinical presentation, in line with the Brazilian literature, which indicates a prevalence between 46% and 92%,^4^ but contrasts with the findings of a study carried out in 704 children with sporotrichosis in China, where the main route of transmission was environmental and there was a predominance of the fixed cutaneous form.^2^ With the advancement of the zoonotic epidemic caused by *S. brasiliensis*, presentations previously considered atypical have been on the rise, with immunoreactive forms such as erythema nodosum and Sweet's syndrome being reported more frequently in the context of the current epidemic.^1^, ^3^, ^4^ Lesions in the ocular mucosa, for example, have become more frequent and are associated with exposure to aerosols from infected animals, autoinoculation after contact with fomites, or direct contact with the animals. Similarly, multiple inoculation in different cutaneous regions has been observed as a consequence of recurrent trauma (bites and scratches) common in childhood interactions with infected cats.^4^

The main vectors of zoonotic sporotrichosis transmission are infected felines, which have a high fungal load in their lesions.^3^ In the present study, direct contact with sick cats was identified as the main route of exposure, and 42% of owners reported the loss or death of the animal. These data reinforce the impact of the abandonment of infected cats, also evidenced by Chaves et al.,^7^ who observed an abandonment rate of 21% among 147 monitored cats, of which 54.4% died. Such evidence highlights the urgency of public policies that ensure the monitoring and proper veterinary management of these animals, a fundamental measure for controlling the epidemiological chain of sporotrichosis and for interrupting the infection in both humans and animals.

Sabouraud Dextrose Agar medium was used as the culture medium, considered the gold standard for the diagnosis of sporotrichosis.^4^ It showed a positive result in 75% of the evaluated cases. Although it demonstrates high sensitivity, this performance does not reach the 95.2% sensitivity observed in cases of feline sporotrichosis, a difference attributed to the lower fungal load in human samples, clinical variability, or technical limitations in collection.^8^ In this context, histopathological examination plays a complementary role in diagnostic investigation, with suggestive findings such as suppurative granulomas and granulomatous dermatitis.^2^, ^3^, ^4^ In the present study, the examination revealed alterations compatible with sporotrichosis in 57.8% of cases. However, the visualization of yeasts using the special PAS and Grocott stains was possible in only 21% of cases with histopathological analysis (4/19). This limitation is expected and reflects the low sensitivity of this isolated examination, which varies between 18% and 35% in the literature, depending on the fungal load and the technique employed.^2^ Nevertheless, the identification of fungal structures in two cases with negative cultures reinforces the relevance of histopathological examination as a complementary diagnostic tool, especially when culture fails, contributing to the clarification of challenging cases.

The predominant therapeutic strategy consisted of the administration of itraconazole, a first-line treatment for sporotrichosis, due to its efficacy and the high cure rates (90%–100%) reported in the literature.^4^, ^5^, ^6^, ^7^, ^8^, ^9^, ^10^ In a prospective study that used Potassium Iodide (KI) in patients with sporotrichosis, including a cohort of children (<15 years), 100% cure rates were obtained,^11^ demonstrating an efficacy close to that obtained in this study (81.8%). In addition, KI has the advantage of allowing formulation in syrup form, which facilitates administration in the pediatric population. The average treatment time observed, from three to five months, varied according to the medication used and was consistent with previous studies, which describe an average time for clinical resolution between two and four months.^2^, ^4^, ^11^

Based on the present findings, this study contributes to the recognition of sporotrichosis occurring in the pediatric age group as important in the current urban zoonotic epidemic caused by *S. brasiliensis*. The varied clinical manifestations, diagnostic challenges, and the impact of direct exposure to sick felines reinforce the need for specific protocols for the pediatric age group. In addition, the data highlight the urgency of public strategies focused on veterinary control and integrated surveillance, which are fundamental to containing the transmission chain and mitigating the impact of the disease on vulnerable groups.

## ORCID ID

Guilherme Camargo Julio Valinoto: 0000-0003-3365-0280

John Verrinder Veasey: 0000-0002-4256-5734

## Financial support

None declared.

## Authors' contributions

Bruna Cavaleiro de Macedo Souza: Design and planning of the study; collection of data, or analysis and interpretation of data; drafting and editing of the manuscript or critical review of important intellectual content; acquisition, analysis, and interpretation of data; critical review of the literature; approval of the final version of the manuscript.

John Verrinder Veasey: Design and planning of the study; collection of data, or analysis and interpretation of data; drafting and editing of the manuscript or critical review of important intellectual content; acquisition, analysis, and interpretation of data; effective participation in research orientation; intellectual participation in the propaedeutic and/or therapeutic conduct of the studied cases; critical review of the literature; approval of the final version of the manuscript.

Guilherme Camargo Julio Valinoto: Intellectual participation in the propaedeutic and/or therapeutic conduct of the studied cases; approval of the final version of the manuscript.

## Research data availability

The entire dataset supporting the results of this study was published in this article.

## Conflicts of interest

None declared.
